# The Pathology and Treatment of Sun Stroke

**Published:** 1869-04

**Authors:** George Johnson


### SELECTIONS.

The Pathology and Treatment of Sunstroke.—The formidable disease known by the name of *sunstroke* or *heat-apoplexy*, might (Dr. Johnson writes) be more correctly designated *heat-apnœa.* Although this affection frequently occurs from direct exposure to the sun's rays, it is also of common occurrence without such exposure. The one essential and constant condition is a very high temperature of the air. The most powerful concurring causes are—muscular exertion and excessive fatigue; hot clothing, and especially such as tends to impede the respiratory movements; an excessive use of alcoholic liquors; and the close and impure air of hot and overcrowded rooms. The disease may be fatal in a few minutes, or the symptoms may last from one to forty-eight hours.

The rapidly fatal cases are spoken of as belonging to the *cardiac* variety. The patient falls unconscious, gasps, and dies. When the disease runs a less rapid course, it is said to be of the *cerebro-spinal* variety. There are great heat, dryness, and redness of the skin, giddiness, nausea, congestion of the eyes, and frequent desire to micturate; sometimes delirium, then drowsiness, passing into coma. The pupils are contracted; the breathing is hurried and laborious; the heart's action is tumultuous; the pulse rapid, at first distinct, but soon becoming feeble and irregular. Convulsions are of common occurrence, either early in the attack, or immediately before death. After death, however rapid may have been the course of the disease, the one constant condition is extreme, "unexampled" congestion of the lungs, with distention of the right side of the heart.

Dr. Maclean, to whose article on Sunstroke (Reynold's *Sys-*

*tem of Medicine*) Dr. Johnson would refer for a clear and succinct account of the facts of the disease, states that all modern pathologists are agreed that the superheating of the blood, which precedes and accompanies sunstroke, has a depressing, and not a stimulating, effect on the nervous centres. In what way, then, does the overheated blood exert this depressing effect on the nervous centres? Dr. Johnson believes the following to be the true physiological explanation of the phenomena.

The hot blood relaxes the muscular walls of the minute pulmonary arteries. The pulmonary capillaries are consequently flooded with blood. This over-fulness of the capillaries interferes with the aeration of the blood. In fact, the over-gorged vessel must encroach upon the pulmonary vesicles, and so diminish the air-space within the lungs; while the air itself is highly rarefied. Hence a state of more or less complete apnœa. Unaerated blood is sent to the muscular tissue of the heart, and to the brain: hence the cardiac and the cerebral symptoms. A similar engorged state of the cutaneous capillaries, consequent upon extreme relaxation of the minute arteries, is the probable cause of the dryness of the skin. An excessively engorged state of the capillaries is as unfavorable for cutaneous secretion as it is for pulmonary respiration. The dry and inactive state of the skin and the want of surface-evaporation tend to elevate still more the temperature of the blood; and the suppressed cutaneous secretion, being diverted to the kidneys, probably alters the quantity of the urine, renders it irritating to the bladder, and explains the frequent micturition.

This explanation of the phenomena is confirmed by the results of treatment. There is now a very general concurrence of opinion that the application of cold to the skin is the most successful remedy. The object to be kept in view is not merely, as it is generally stated, to cool the skin, or to excite the respiratory movements by the stimulus of the douche, but to cool the blood, and thus to restore the contractility of the minute arteries of the lungs. The condition of the pulmonary vessels in this disease is the exact opposite to their state in cholera collapse. In cholera collapse, the minute pulmonary arteries are in a state of extreme contraction; and as a consequence, the capillaries are extremely anæmic. In heat-apnœa the pulmonary arteries are extremely relaxed; and the capillaries, consequently, are excessively engorged. In cholera collapse, external warmth in some degree, but much more rapidly and decidedly a warm injection into the veins, relaxes the arterial spasm, and restores the circulation. In heat-apnœa, on the contrary, the object is to cool down the overheated blood, so to revive the contractile power of the minute pulmonary arteries, to relieve the capillaries from their embarrassing excess of blood, and thus to remove the state of apnœa. A clear apprehension of these physiological principles cannot fail to be of great assistance in practice.

In the treatment of heat-apnœa the following appear to be the main points which require attention. The patient should be placed in a recumbent position in the coolest possible place, with a free current of air. The clothes should be removed, and cold water applied to the whole surface ; or if the symptoms be urgent, the clothes should immediately be saturated with cold water without waiting to remove them. If the respiratory movements be failing and feeble, the cold douche is a powerful excitor ; but if the breathing be rapid and laborious, it is better to envelope the body in a wet sheet, and to quicken evaporation and cooling by a fan or a pair of bellows. If the patient can swallow, let him drink iced water freely. Whether he can swallow or not, iced water may from time to time be injected. The marvelous effect of hot venous injections in cholera collapse, and the urgent need for cooling the blood in heat-apnœa, suggest the expediency, in extreme cases, of injecting into the vein the same saline solution as has so frequently been employed in cholera, only injecting it cold instead of hot.

A routine practice of venesection would be destructive ; but when symptoms of excessive venous engorgement are present, a cautious venesection would be quite justifiable, and probably beneficial, on the well-known principle of lessening distension of the right side of the heart, and thus increasing its contractile power. When respiration has suddenly and quite recently ceased, artificial respiration by Dr. Silvester's method may possibly restore animation. While symptoms of apnœa continue, however great may be the apparent exhaustion, no alcoholic stimulants are to be given, for the reason that alcohol, as well as anæsthetic vapors and narcotics, impede oxidation of the nervous and other tissues, and therefore increase the risk of death from apnœa. Ammonia may be applied to the nostrils as a stimulant, and, if the patient can swallow, it may be given internally. Ammonia is a powerful diaphoretic, and the restoration of the cutaneous secretion is an important step towards recovery. When the skin becomes cool and moist, of course all cold applications are to be discontinued. To sum up then—as *hot air* and *hot blood* are the cause of this form of apnœa, so *cold air* and *cold water* are the chief means of cure ; all other means are subsidiary to these.—*British Medical Journal*, *August* 1*st*, *by* GEORGE JOHNSON, M. D., F. R. C. P.

